# Electromagnetic Shielding Effectiveness of Woven Fabrics with High Electrical Conductivity: Complete Derivation and Verification of Analytical Model

**DOI:** 10.3390/ma11091657

**Published:** 2018-09-07

**Authors:** Marek Neruda, Lukas Vojtech

**Affiliations:** Department of Telecommunication Engineering, Faculty of Electrical Engineering, Czech Technical University in Prague, Technicka 2, 166 27 Prague, Czech Republic; lukas.vojtech@fel.cvut.cz

**Keywords:** analytical model, electromagnetic shielding effectiveness, electric properties, fabric, woven textiles

## Abstract

In this paper, electromagnetic shielding effectiveness of woven fabrics with high electrical conductivity is investigated. Electromagnetic interference-shielding woven-textile composite materials were developed from a highly electrically conductive blend of polyester and the coated yarns of Au on a polyamide base. A complete analytical model of the electromagnetic shielding effectiveness of the materials with apertures is derived in detail, including foil, material with one aperture, and material with multiple apertures (fabrics). The derived analytical model is compared for fabrics with measurement of real samples. The key finding of the research is that the presented analytical model expands the shielding theory and is valid for woven fabrics manufactured from mixed and coated yarns with a value of electrical conductivity equal to and/or higher than *σ* = 244 S/m and an excellent electromagnetic shielding effectiveness value of 25–50 dB at 0.03–1.5 GHz, which makes it a promising candidate for application in electromagnetic interference (EMI) shielding.

## 1. Introduction

Electromagnetic compatibility is the branch of electrical engineering focused on generation, propagation, and reception of electromagnetic energy that can affect the proper function of electronic systems. One of the methods for ensuring proper function of these systems is a shielding, expressed by a quantity called shielding effectiveness (SE), electromagnetic shielding, or electromagnetic shielding effectiveness. Primarily, the shielding of electronic systems is performed by metals. Nowadays, the metals can be replaced by electrically conductive textiles in order to obtain a relevant value of the SE, which has been a highly discussed topic in recent years. The structure of these textile materials can be in the form of coated/metallized fabric, which can be categorized as a multi-layered “stack-up” system of composite shielding materials, or particulate-blended shielding textile composites, which are made up by metallic inclusions like aluminum, copper, silver, or nickel particles heterogeneously mixed in a host medium such as polymer/plastic. The main benefits include lower consumption of metals, flexibility of the textile materials, mechanical properties, and/or lower weight of the shielding. Woven fabrics with high electrical conductivity are being increasingly utilized in the shielding of electromagnetic interference (EMI) and in electrostatic protection in various applications such as the shields for equipment cases, the protective clothing for personnel working under high-voltage magnetic fields and/or in radiofrequency/microwave environments, shielding and grounding curtains, electrostatic discharge wipers, flexible shielded shrouds, smocks, stockings, boots, etc.

Many research papers describe SE evaluation from different perspectives, i.e., measurement techniques [1,2,3,4,5,6,7], composition of materials [8,9,10,11,12,13,14,15,16,17], influence of washing/drying cycles on values of SE of fabrics [18,19,20], or calculation of SE [4,21,22,23,24,25,26,27,28,29,30,31]. SE measurement is commonly performed by a coaxial transmission line method specified in ASTM 4935-10 [1,2,3,4,6,8,9,10,12,14,17,18] by measuring the insertion loss with a dual transverse electromagnetic (TEM) cell [3,5], or by measurement in a free space, shielding box, or shielding room with receiving and transmitting antennas [7,15,19,20]. The papers which focus on measurement techniques of various electrically conductive fabrics usually present basic equations for SE calculation [4,21,22,23,24,25,26,27,28] (SI units are used in all formulas unless otherwise stated) as shown in Equation (1).

(1) $$SE=20\cdot \log_{10}\left|\frac{E_{i}}{E_{t}}\right|\;=20\cdot \log_{10}\left|\frac{H_{i}}{H_{t}}\right|=10\cdot \log_{10}\left|\frac{P_{i}}{P_{t}}\right|\;=R+A+B\;$$

where *E_i_*, *H_i_*, and *P_i_* are the electric field intensity, magnetic field intensity, and power without the presence of tested material (incident electromagnetic field on the tested material), respectively, *E_t_*, *H_t_*, and *P_t_* are the same physical quantities with the presence of tested material (transmitted electromagnetic field measured behind the tested material), *R* is the reflection loss, *A* is the absorption loss, and *B* is multiple reflections.

Reflection loss *R* (also called return attenuation) is a consequence of the electromagnetic wave reflection on the interface. The absorption loss *A* (also called absorption attenuation) is produced if the electromagnetic wave is transferred through the shielding barrier. A portion of energy is absorbed in the shielding barrier due to heat loss. Attenuation caused by multiple reflections, *B*, is physically caused by electromagnetic wave propagation in the conducted shielding barrier. The electromagnetic wave is repetitively reflected on the “inner” interfaces of the material.

The handbook of electromagnetic materials [28] describes an expression for SE calculation of metallized fabrics based on transmission line theory, i.e., an analysis of the leakage through apertures in the fabric, as shown in Equation (2):

(2) $$SE=A_{a}+R_{a}+B_{a}+K_{1}+K_{2}+K_{3}\;$$

where *A_a_* is the attenuation introduced by a particular discontinuity, *R_a_* is a fabric aperture with single reflection loss, *B_a_* is the multiple reflection correction coefficient, *K*_1_ is the correction coefficient to account for the number of like discontinuities, *K*_2_ is the low-frequency correction coefficient to account for skin depth, and *K*_3_ is the correction coefficient to account for a coupling between adjacent holes.

The authors in [4] adopted this formula without description of its derivation for hybrid fabrics, i.e., fabrics composed of hybrid yarns containing polypropylene and different content. This formula is also compared with the wave-transmission-matrix (WTM) method in [8]. The authors evaluated the SE of the laminated and anisotropic composites for single-layer and multi-layer fabrics. The same formula is also presented in [23] for evaluation of copper core-woven fabrics in order to identify dependencies of the SE on the material structure. None of the papers [4,8,23] nor the handbook [28] present a derivation of this formula. The influence of seaming stitches on the SE fabric is described in [29]. That paper presents a computation model of the SE based on the equivalent seaming gap. Analytical formulation for the SE of enclosures with apertures is described in [30]. The paper presents an extended theory to account for electromagnetic losses, circular apertures, and multiple apertures. Formulas for the apertures (especially multiple apertures) are key to the analytical modeling of fabrics. A calculation method of SE for woven fabric containing metal fiber yarns is deduced through the transfer matrix of the electromagnetic field numerical calculation in [31].

A semi-empirical model describing the plane wave SE for fabrics is presented in [25]. The authors focus only on coated fabrics and derivation of the SE formula based on electrical properties (especially electrical conductivity). The same formula is also described in the handbook [28] without a formula derivation, Equation (3).

(3) $$SE_{fabric}=e^{-0,129\cdot \ell \cdot \sqrt{f}}\cdot SE_{foil}+\left(1-e^{-0,129\cdot \ell \cdot \sqrt{f}}\right)\cdot SE_{aperture}\;$$

where *SE_foil_* and *SE_aperture_* are the SE values for metallic foil (of the same thickness as the fabric) and for the same foil with aperture (s), *l* is the aperture size of the fabric, and *f* is the frequency. 

Calculation of the *SE_foil_* is well-known from shielding theory [24,26,27,28,32,33] as:

(4) $$SE_{foil}=\underset{R_{foil}}{\underset{︸}{20\cdot \log\left|\frac{{\left(Z_{0}+Z_{M}\right)}^{2}}{4Z_{0}Z_{M}}\right|}}+\underset{A_{foil}}{\underset{︸}{20\cdot \log\left|e^{t/\delta }e^{-j\beta_{0}t}e^{j\beta t}\right|}}+\underset{B_{foil}}{\underset{︸}{20\cdot \log\left|1-e^{-2t/\delta }e^{-j2\beta t}{\left(\frac{Z_{0}-Z_{M}}{Z_{0}+Z_{M}}\right)}^{2}\right|}}\;$$

where *Z*_0_ is the impedance of free space, *Z_M_* is the impedance of shielding barrier, *t* is the thickness, *δ* is the penetration depth, *β*_0_ is the vacuum phase constant, and *β* is the phase constant.

A complete derivation of Equation (4) is published in our previous research papers [26,27]. Calculation of *SE_aperture_* is usually expressed similarly to Equation (1), and it is expressed only for metallized fabric shields as [25,28], Equation (5).

(5) $$SE_{aperture}=R_{aperture}+A_{aperture}+K_{aperture}=100-20\cdot \log\left(L\cdot f\right)+20\cdot \log\left(1+\ln\left(\frac{L}{s}\right)\right)+30\frac{D}{L}\;$$

where *L* is the maximum aperture size, *f* is the frequency of operation, *s* is the minimum aperture size, and *D* is the depth of the aperture.

Calculation and derivation of *SE_aperture_* is not present in the scientific literature for particulate-blended shielding electrically conductive textile composites. Therefore, the main contribution of this this paper is that the research performed a complete derivation of an analytical model of SE for woven textile materials manufactured from electrically conductive mixed and coated yarns, i.e., for particulate-blended shielding electrically conductive textile composites. Basic simplifications, which are valid for metals, were also evaluated for these textile materials. A complete derivation of SE evaluation was also performed for *SE_fabric_* (Equation (3)) and *SE_aperture_* of metallized fabric shields (Equation (5)). A general equation for SE evaluation for particulate-blended shielding electrically conductive textile composites with electrical conductivity bigger than 244 S/m was derived and compared with measurement of real samples according to ASTM 4935-10. The maximal difference between modeling and measurement results was in the range of 2–6 dB, which is within the random error of the used measurement method, i.e., ±5 dB.

## 2. Experimental Materials

The samples are particulate-blended shielding electrically conductive textile composites manufactured from two types of yarns that are mixed and coated with a plain weave fabric structure, Table 1. The coated yarns SilveR.STAT^®^ (samples #1–#2) contain a very pure silver layer on the polymer base (Polyamide). The mixed yarns (samples #3–#7) are blended from the non-conductive textile material, i.e., Polyester (PES), and the conductive material, i.e., silver in the form of the SilveR.STAT^®^ coated yarns. The plain weave is chosen because of its simple and regular structure.

The samples #1 and #2 and #3–#5 are made of the same material (the same ratio of conductive and non-conductive textile material in the case of #3–#5) with the same fabric structure and differ from each other mainly by the warp and weft density used in a production process. The samples #6 and #7 are characterized by the same warp and weft density, the same fabric structure, and differ from each other by the ratio of conductive and non-conductive textile material, i.e., 40%/60% and vice versa. The selected parameters result in a different value of mass per unit area and, more importantly, in the different electrical conductivity value. As a result, the three groups of electrically conductive textile materials can be distinguished by the value of the order of the electrical conductivity, i.e., #1–#2, #3–#5 and #6–#7, which is an important parameter in the SE calculation as shown in the equations, e.g., Equations (4) and (6).

Measurement of the electrical conductivity of samples #1–#7 is based on a four electrode test method described in BS EN 16812:2016 [34] and conclusions presented in [35], i.e., measurement of surface and bulk resistance is equal for high electrically conductive textile materials and, therefore, thickness of the sample can be taken into account in electrical conductivity evaluation. Mean value (evaluated for five different lengths and five different areas of the sample, 65% RH, 20 °C) and standard deviation of electrical conductivity are depicted in Table 1.

## 3. Evaluation of Reflection Loss of Foil

Reflection loss *R_foil_* is generally expressed in Equations (4) and (6). It can be simplified for metals because of its good electrical conductivity, i.e., the inequality *Z_M_* << *Z*_0_ is valid. Moreover, the impedance of the material *Z_M_* is further simplified because of the validity *σ* >> *ωε*. The *R_foil_* is then calculated as [26,27], Equation (6).

(6) $$R_{foil}=20\cdot \log\left|\frac{{\left(Z_{0}+Z_{M}\right)}^{2}}{4Z_{0}Z_{M}}\right|\approx 20\cdot \log\left|\frac{Z_{0}}{4Z_{M}}\right|=20\cdot \log\left|\frac{\sqrt{\frac{\mu_{0}}{\varepsilon_{0}}}}{4\sqrt{\frac{j\omega \mu }{\sigma +j\omega \varepsilon }}}\right|\approx 20\cdot \log\left|\frac{\sqrt{\frac{\mu_{0}}{\varepsilon_{0}}}}{4\sqrt{\frac{\omega \mu }{\sigma }}}\right|=20\cdot \log\left(\frac{1}{4}\sqrt{\frac{\sigma }{\omega \mu_{r}\varepsilon_{0}}}\right)\;$$

where *µ*_0_ is the vacuum permeability, *µ_r_* is the relative permeability, *µ* is the permeability of a specific medium, *ω* is the angular speed, *σ* is the conductivity, *ε*_0_ is the vacuum permittivity, and *ε* is the absolute permittivity.

The conductivity of a material can be expressed as conductivity relative to copper [28]. The value of copper conductivity is equal to *σ_Cu_* = 5.8 × 10^7^ S/m [33,36]. Material conductivity is described as *σ* = *σ_r_σ_Cu_*, and *R_foil_* is expressed as shown in Equation (7).

(7) $$R_{foil}=20\cdot \log\left(\frac{1}{4}\sqrt{\frac{\sigma_{Cu}}{2\pi \varepsilon_{0}}}\right)+20\cdot \log\left(\sqrt{\frac{\sigma_{r}}{f\mu_{r}}}\right)=168.14+20\cdot \log\left(\sqrt{\frac{\sigma_{r}}{f\mu_{r}}}\right)\;$$

where *f* is the frequency of the operation.

A similar equation can be also found in [4,21,22,24]. The calculation of reflection loss *R_foil_* corresponds to the copper conductivity value, e.g., *σ*_Cu_ = 5.82 × 10^7^ S/m [32], 5.7 × 10^7^ S/m [37], or 5.85 × 10^7^ S/m [38], which depends on the purity and the production method of copper. Nevertheless, Equation (7) is presented for fabrics, i.e., the value *σ_Cu_* = 5.8 × 10^7^ S/m is used. This means the authors presume the validity of presented inequalities, i.e., *Z_M_* << *Z*_0_ and *σ* >> *ωε*. This presumption is furthermore verified. Definitions of the impedances *Z_M_* and *Z*_0_ and their simplified versions are described in Equation (8).

(8) $$Z_{0}=\sqrt{\frac{\mu_{0}}{\varepsilon_{0}}}=\sqrt{\frac{4\cdot \pi \cdot 10^{-7}}{8,854\cdot 10^{-12}}}=120\;\pi \approx 377\;Z_{M}=\sqrt{\frac{j\omega \mu }{\sigma +j\omega \varepsilon }}\;\approx \sqrt{\frac{\omega \mu }{\sigma }}\;$$

**Figure 1:**
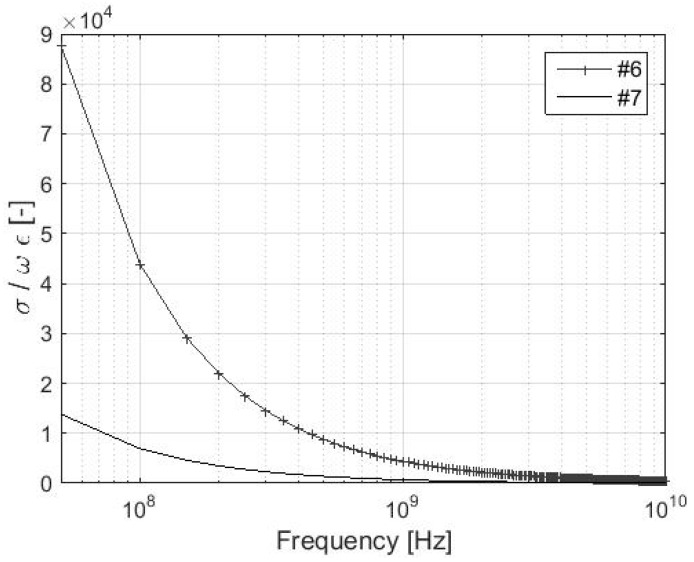
Ratio *σ*/*ωε* for samples #6 and #7.

**Figure 2:**
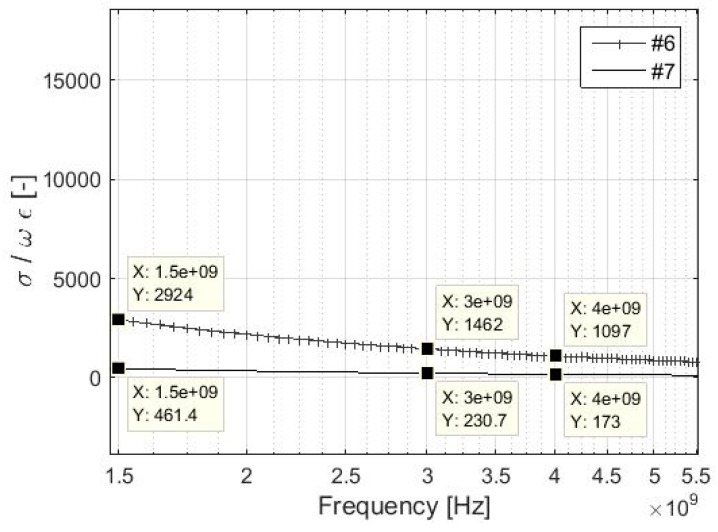
Details of the ratio *σ*/*ωε* for samples #6 and #7 in 1.5–5.5 GHz.

The validity of the *σ* >> *ωε* can be easily verified for the lowest values of the electrical conductivity of the samples, i.e., #6 and #7. The value of relative permittivity of the used electrically conductive material is considered to be *ε_r_* = 1, because the non-conductive textile material is blended with a conductive material, i.e., silver, Table 1. As a consequence, the resultant textile material is categorized as lossy conductive material, which can be characterized as *ε_r_* = 1. The value of relative permeability is considered to be equal to *µ_r_* = 1. Results for different frequencies are presented in Table 2, and Figure 1 and Figure 2. The condition of *σ* >> *ωε* is fulfilled for sample #6 in the entire analyzed frequency range, i.e., 30 MHz–10 GHz because of the difference between the values of *σ* and *ωε* in at least two orders of magnitude. Sample #7 fulfills the condition up to approximately 6.9 GHz. As a consequence, a simplified version of the *Z*_M_ and Equation (7) can be used for #1–#6 up to 10 GHz and for #7 up to approximately 6.9 GHz. Materials with lower electrical conductivity than #7, i.e., 39 S/m, have to be analyzed in order to obtain the frequency limit of validity *σ* >> *ωε* and Equation (7). It can also be noted the limit of #6 is found to be 43.85 GHz, and the SE measurement is usually performed by a coaxial transmission line method specified in ASTM 4935-10 in the range of 30 MHz–1.5 GHz [39].

**Figure 3:**
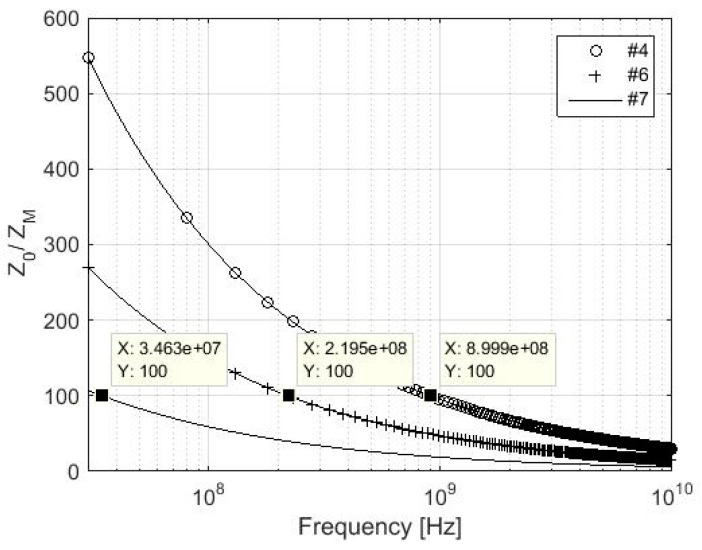
Ratio *Z*_0_/*Z_M_* for samples #4, #6, and #7; *σ* >> *ωε* is not assumed.

**Figure 4:**
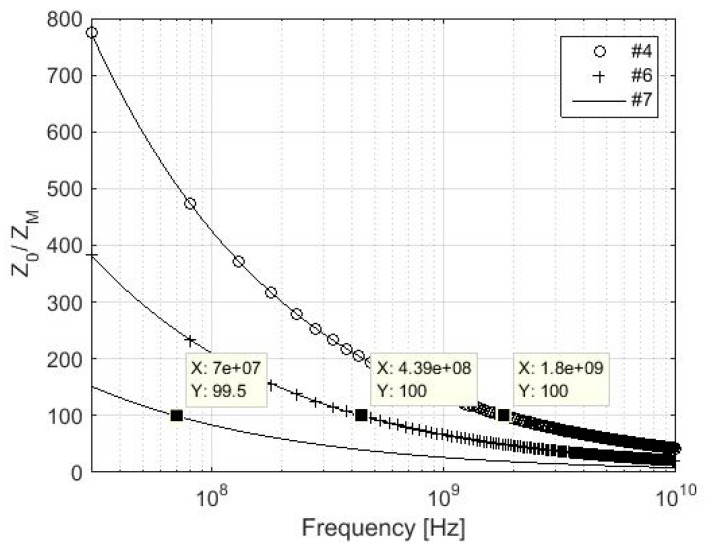
Ratio *Z*_0_*/Z_M_* for samples #4, #6, and #7; *σ* >> *ωε* is assumed.

The validity of *Z_M_ << Z*_0_ is verified for samples #7, #6, and #4, which are characterized by lower values of electrical conductivity from all described samples, Figure 3 and Figure 4. The validity of *σ* >> *ωε* is assumed, i.e., a simplified version of *Z_M_* is considered. A difference in values of two orders of magnitude in the frequencies 70 MHz, 439 MHz, and 1.8 GHz can be seen for #7, #6, and #4, respectively. If the validity of *σ* >> *ωε* is not assumed, the values of two orders of magnitude are in the frequencies 34.6 MHz, 219.5 MHz, and 899.9 MHz for #7, #6, and #4, respectively.

**Figure 5:**
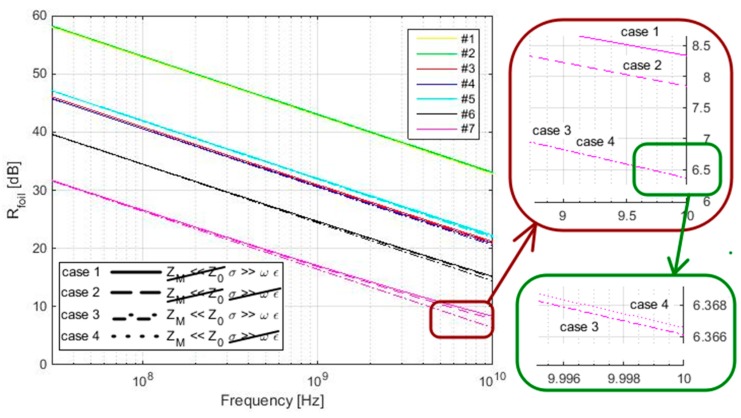
*R_foil_* evaluation for #1–#7 and *Z_M_ << Z*_0_ and *σ* >> *ωε* are/are not considered (four cases).

The clarity of application of the validity *Z_M_ << Z*_0_ and *σ* >> *ωε* can be also seen in Figure 5. It compares the *R_foil_* parameter of the four cases, i.e., *Z_M_ << Z*_0_ and *σ* >> *ωε* are/are not considered (in all four cases), and also for all samples #1–#7. All four cases are almost identical to #1–#5, i.e., the greatest difference is reached for the sample with the lowest electrical conductivity (#4), and it is equal to about 0.5 dB in 10 GHz. The results also show an insignificant difference, i.e., the greatest difference is about a thousandth of a dB, for cases *Z_M_ << Z*_0_ with (dash-dot line) and without (dotted line) consideration of *σ* >> *ωε* validity for the sample with the lowest electrical conductivity (#7) (case 3 and case 4). The greatest difference between these four cases is obtained for #7 in the frequency 10 GHz. It is about 2 dB for the cases where *Z_M_ << Z*_0_ is not considered and *σ* >> *ωε* is considered (solid line) and where *Z_M_ << Z*_0_ is considered and *σ* >> *ωε* is (dash-dot line) / is not (dotted line) considered (both cases of *σ* >> *ωε* show similar results as previously mentioned) (case 1 and case 3). The same situation is also valid for #6 with a difference not exceeding 1 dB. The results also show higher values of *R_foil_* for #7 at 10 GHz, i.e., about 0.5 dB, for the case where *Z_M_ << Z*_0_ is not considered and *σ* >> *ωε* is considered (solid line) in comparison with the case where *Z_M_ << Z*_0_ and *σ* >> *ωε* are not considered (dashed line) (case 1 and case 2), i.e., no simplification is performed.

As a consequence, a simplification of SE evaluation by *Z_M_ << Z*_0_ and *σ* >> *ωε* in frequency range up to 10 GHz is valid for samples with electrical conductivity values higher than 1000 S/m with an error up to 0.5 dB, for samples with an electrical conductivity value of 244 S/m with an error not exceeding 1 dB and for samples with an electrical conductivity value of 39 S/m and an error up to 2 dB.

## 4. Evaluation of Reflection Loss of Aperture

Reflection loss of aperture *R_aperture_* is generally derived from the basic relation between the gain and effective aperture of the antenna. It is described as [33], Equation (9).

(9) $$A_{e}=\frac{\lambda^{2}}{4\pi }G\;$$

where *λ* is wavelength, *A_e_* is effective aperture and, *G* is the gain.

The parameter *A_e_* differs for different shapes of antenna loops [40]. Woven textile materials are manufactured by interlacing the yarns at right angles and, therefore, a rectangular or square shape of the apertures can be considered. Presented samples are manufactured from the same yarn and by the same sett in the warp and weft directions. As a consequence, the aperture is square shaped. For the square shape of the antenna loop, the effective aperture is calculated as [40], Equation (10).

(10) $$A_{e}=l^{2}\;$$

where *l* is the length of the aperture. 

The equality of Equations (9) and (10) describes the gain in Equation (11).

(11) $$G_{aperture\_square}=\frac{4\pi \ell^{2}}{\lambda^{2}}={\left(\frac{2\ell \sqrt{\pi }}{\lambda }\right)}^{2}\;$$

The equation for *G* calculation for the circular loop antenna and for a slot is commonly mentioned in the literature as [33], Equations (12) and (13).

(12) $$G_{aperture\_circular}={\left(\frac{2\pi r}{\lambda }\right)}^{2}\;$$

where *r* is the radius of the circular loop.

(13) $$G_{aperture\_slot}={\left(\frac{2l}{\lambda }\right)}^{2}\;$$

where *l* is the length of the slot.

Reflection loss *R_aperture_* is then calculated [33,37] as:

(14) $$R_{aperture\_square}=10\cdot \log\left(\frac{1}{G_{aperture\_square}}\right)=20\cdot \log\left(\frac{\lambda }{2l\sqrt{\pi }}\right)\;$$

(15) $$R_{aperture\_circular}=10\cdot \log\left(\frac{1}{G_{aperture\_circular}}\right)=20\cdot \log\left(\frac{\lambda }{2\pi r}\right)\;$$

(16) $$R_{aperture\_slot}=10\cdot \log\left(\frac{1}{G_{aperture\_slot}}\right)=20\cdot \log\left(\frac{\lambda }{2l}\right)\;$$

Equations (14)–(16) can be also described as:

(17) $$R_{aperture\_square}=20\cdot \log\left(\frac{c}{2\sqrt{\pi }}\right)-20\cdot \log\left(\ell \cdot f\right)=158.55-20\cdot \log\left(\ell \cdot f\right)\;$$

(18) $$R_{aperture\_circular}=20\cdot \log\left(\frac{c}{2\pi }\right)-20\cdot \log\left(r\cdot f\right)=153.58-20\cdot \log\left(r\cdot f\right)\;$$

(19) $$R_{aperture\_slot}=20\cdot \log\left(\frac{c}{2}\right)-20\cdot \log\left(l\cdot f\right)=163.52-20\cdot \log\left(l\cdot f\right)\;$$

where *c* is the speed of light.

Equations (17)–(19) are derived for one aperture in the foil. It can be seen that they are valid for any material with an aperture, because reflection loss of aperture *R_aperture_* is dependent on the frequency and dimensions of an aperture.

## 5. Evaluation of Reflection Loss of Multiple Apertures

Multiple apertures are discussed in [32,36,37]. The equation for multiple apertures is described as:

(20) $$R_{aperture\_multiple}=-20\cdot \log\sqrt{n}\;$$

where *n* is the number of apertures.

Nevertheless, calculation of the number of apertures *n* is not unified in [32,36,37]. The conditions for validity of Equation (20) follow:

- Reference [32]: linear array of apertures, equal sizes, closely spaced apertures, and the total length of linear array of apertures is less than ½ of the wavelength. If the two-dimensional array of holes is considered, Equation (20) can be directly applied only for the first row of apertures (the rest of the apertures are not included in parameter *n*). This means, if the two-dimensional array is given by 7 × 12 holes, then *n* = 12. This approximation is motivated by experience.
- Reference [36]: equally sized perforations, hole spacing < *λ*/2, hole spacing > thickness, *n* is the number of all apertures.
- Reference [37]: thin material, equally sized apertures, *n* is the number of all apertures.

**Figure 6:**
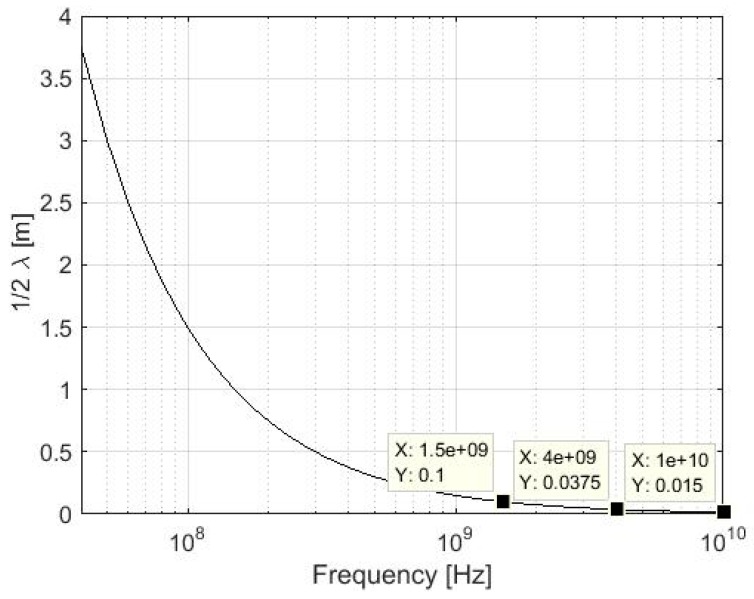
Calculation of the ½ wavelength.

The minimal value of the wavelength can be easily found as depicted in Figure 6. 

**Figure 7:**
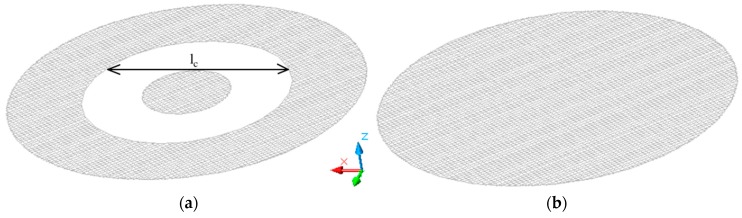
The longest linear array of apertures found in the reference (**a**) and load sample (**b**) according to ASTM 4935-10 (the white annulus of (**a**) matches the size of the measured sample).

The longest linear array of apertures can be determined with respect to ASTM D4935-10. The standard ASTM D4935-99 was withdrawn in 2005, because the committee could not maintain a standard for which the expertise did not lie within the current committee membership [41]. It also describes the dimensions of the measured samples. The longest linear array of apertures that can be found on the sample is the tangent of the inner circle limited by the middle circle. It is indicated by the double arrow with the parameter *l_c_* in Figure 7a. It shows the shape of the reference sample, which matches the size of the sample holder, i.e., the measured part of the sample corresponds to the white annulus in Figure 7a. The distance is equal to *l_c_* = 0.069 m, i.e., the total length of apertures is less than ½ of the wavelength at 0.03–1.5 GHz. Figure 7b shows the load sample.

The apertures are equally sized, closely spaced, and form a linear array of apertures because of the production process of textiles and parameters used during the production of samples.

The textile structure forms the two-dimensional array of holes and the longest linear array of apertures is equal to *l_c_* = 0.069 m.

Hole spacing is equal to the yarn diameter, which is in the range of 0.220–0.251 × 10^−3^ m. It is less than ½ of the wavelength, and it is less than the thickness of the material, which is at a minimum equal to 0.295 × 10^−3^ m, Table 3.

**Figure 8:**
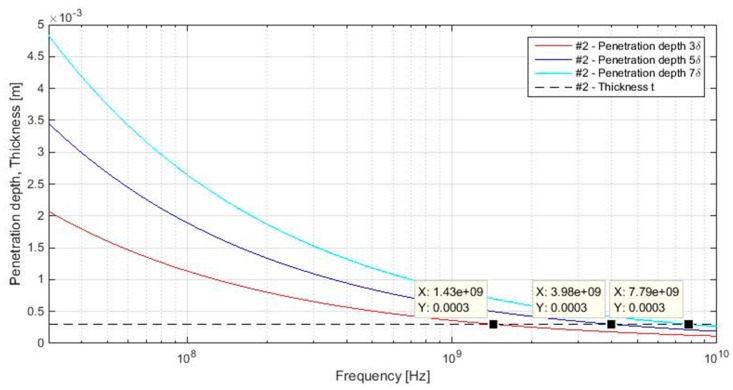
Penetration depths 3*δ*, 5*δ,* and 7*δ* in comparison with thickness for #2.

A difference of the thin and thick material is presented in [36]. The material is considered to be thick when there is no reflection from the “far” interface of the material. This definition can be verified by the equivalent depth of penetration *δ*, Equation (21), which defines a distance of wave penetration to amplitude wave degradation to the value e^−1^, i.e., amplitude wave degradation of about 36.8% in comparison with the thickness of the material. If we consider 3*δ*, amplitude wave degradation is about 95%, i.e., 95% of the current flows within a material. This is the point beyond which current flow is negligible in a material [25]. Nevertheless, comparison for almost 100% of amplitude wave degradation can be performed. The penetration depth 4*δ* decreases the amplitude wave to about 98.2%, 5*δ* to about 99.3%, 6*δ* to about 99.8%, and 7*δ* to about 99.9%. If the penetration depths 3*δ*, 5*δ,* and 7*δ* are calculated for sample #2 (the sample with the highest value of electrical conductivity, i.e., the value of penetration depth is the lowest), the dependence for the frequency band 30 MHz–10 GHz is obtained, Figure 8. The results show the penetration depths 3*δ*, 5*δ,* and 7*δ* are lower than the thickness of #2 in the frequency range 1.43–10 GHz, 3.98–10 GHz, and 7.79–10 GHz, respectively. This means that in this frequency range there is no reflection from the “far” interface of the material. In other words, the material is considered to be thick. In the frequency ranges 30 MHz–1.43 GHz for 3*δ*, 30 MHz–3.98 GHz for 5*δ,* and 30 MHz–7.79 GHz for 7*δ*, there are reflections from the “far” interface of the material and therefore the material is considered to be thin, Table 3.

(21) $$\delta =\sqrt{\frac{2}{\omega \mu \sigma }}=\frac{1}{\sqrt{\pi f\mu \sigma }}\;$$

As a consequence of the reflection loss of multiple apertures, Equation (3) has to be specified for the electrically conductive textile samples described, i.e., Equation (20) is added and the values of electrical conductivity of the described samples are considered.

## 6. Evaluation of SE Fabric

An expression for the SE calculation of fabric has been developed on the basis of plane wave shielding theory [25,28]. It is based on a linear combination of the SE of the compact material *f*_1_ (*l, λ*) (in lower frequency ranges) and the SE of the apertures *f***_2_** (*l*, *λ*) (in higher frequency ranges) as shown in Equation (3). It can be written as shown in Equations (22) and (23):

(22) $$f_{1}\left(\ell ,\lambda \right)+f_{2}\left(\ell ,\lambda \right)=1;\;f_{2}\left(\ell ,\lambda \right)=1-f_{1}\left(\ell ,\lambda \right)\;$$

(23) $$SE_{fabric}=f_{1}\left(\ell ,\lambda \right)\cdot SE_{foil}+\left(1-f_{1}\left(\ell ,\lambda \right)\right)\cdot SE_{aperture}\;$$

A one-dimensional base of the solution *f*_1_ (*l*, *λ*), i.e., *f*_1_ (*l*, *f*) with respect to *λ* calculation *λ* = *c*/*f*, can be written as [25], Equation (24).

(24) $$f_{1}\left(\ell ,f\right)=e^{-C\cdot \ell \cdot \sqrt{f}}\;$$

where *C* is the constant.

An assumption of the equality of components, which corresponds to reflection loss *R* of compact material *R_foil_* and material with apertures *R_aperture_*, is used for *f*_1_ (*l*, *f*) derivation, Equation (25).

(25) $$R_{foil}=R_{aperture}\;$$

The parameter *R_foil_* can be used in its simplified version because the *R_foil_* evaluation shows the difference is not significant for samples #1–#6, especially for the frequency range up to 3 GHz, i.e., error does not exceed 0.5 dB, Figure 5. Then Equations (6) and (7) for materials with electrical conductivity *σ* are valid. *R_aperture_* is used from Equations (17) and (20). It is written as:

(26) $$20\cdot \log\left(\frac{1}{4}\sqrt{\frac{\sigma }{\omega \mu_{r}\varepsilon_{0}}}\right)=20\cdot \log\left(\frac{c}{2\sqrt{\pi }}\right)-20\cdot \log\left(\ell f\right)-20\cdot \log\left(\sqrt{n}\right)\;$$

Equation (26) can be modified as shown in Equations (27)–(30).

(27) $$\log\left(\frac{\sigma }{16\omega \mu_{r}\varepsilon_{0}}\right)=\log\left(\frac{c^{2}}{4\pi }\right)-\log\left(\ell^{2}f^{2}\right)-\log\left(n\right)\;$$

(28) $$\log\left(\frac{\sigma }{32\pi f\mu_{r}\varepsilon_{0}}\right)=\log\left(\frac{c^{2}}{4\pi \ell^{2}f^{2}n}\right)\;$$

(29) $$\frac{\sigma n}{8c^{2}\mu_{r}\varepsilon_{0}}=\frac{1}{\ell^{2}f^{}}\;$$

(30) $$l\sqrt{f}=\sqrt{\frac{8c^{2}\mu_{r}\varepsilon_{0}}{\sigma n}}\;$$

A boundary condition, which defines a decrease of the amplitude about 95% in specific material [25], i.e., equivalent of 3 depth of penetration (3*δ* decrease of the amplitude on the multiple e^−1^e^−1^e^−1^ *=* e^−3^ of original value) can be used in Equations (31) and (32).

(31) $$Cl\sqrt{f}=e^{-3}=C\sqrt{\frac{8c^{2}\mu_{r}\varepsilon_{0}}{\sigma n}}\;$$

(32) $$C=\frac{e^{-3}}{l\sqrt{f}}=e^{-3}\sqrt{\frac{\sigma n}{8c^{2}\mu_{r}\varepsilon_{0}}}=1.972\cdot 10^{-5}\sqrt{\sigma n}\;$$

Evaluation of the constant *C* and *n* are shown for samples #1–#7 in Table 4. The number of apertures *n* is calculated with respect to the longest linear array of apertures of ASTM 4935-10, i.e., *l_c_* = 0.069 m and the sett of each sample *d_w_*, Equation (33).

(33) $$n=l_{c}\cdot d_{w}\;$$

As a consequence, Equation (23) is specified for sample #1 as shown in Equation (34).

(34) $$SE_{fabric}=e^{-0.0244\cdot \ell \cdot \sqrt{f}}\cdot SE_{foil}+\left(1-e^{-0.0244\cdot \ell \cdot \sqrt{f}}\right)\cdot SE_{aperture}\;$$

It is obvious the SE calculation has to be specified for each sample with regards to its electrical conductivity, sett, and number of apertures. Therefore, an equation for SE calculation of woven fabrics manufactured from the electrically conductive mixed and coated yarns with square apertures can be written generally with respect to the *C* constant calculation Equation (32) as shown in Equation (35).

(35) $$SE_{fabric}=e^{-C\cdot \ell \cdot \sqrt{f}}\cdot SE_{foil}+\left(1-e^{-C\cdot \ell \cdot \sqrt{f}}\right)\cdot SE_{aperture}\;$$

The calculation of *SE_foil_* is performed according to Equations (4), (7), and (8), and it is also described in depth in [26,27,28,32,33] as shown in Equation (36).

(36) $$SE_{foil}=168.14+20\cdot \log\left(\sqrt{\frac{\sigma_{r}}{f\mu_{r}}}\right)+8.6859\frac{t}{\delta }+20\cdot \log\left|1-e^{-2t/\delta }e^{-j2\beta t}{\left(\frac{Z_{0}-Z_{M}}{Z_{0}+Z_{M}}\right)}^{2}\right|\;$$

### 6.1. Evaluation of SE of Apertures

*SE_aperture_* is calculated as a sum of *R_aperture_*, *A_aperture_,* and *K_aperture_*. *R_aperture_* is derived in this paper and expressed in Equations (17) and (20) as shown in Equation (37).

(37) $$R_{aperture}=158.55-20\cdot \log\left(\ell \cdot f\right)-20\cdot \log\left(\sqrt{n}\right)\;$$

The absorption loss of *A_aperture_* is included in *SE_aperture_* if the fabric is considered to be a thick material, Table 3. It is calculated for a subcritical rectangular waveguide as [32,37], Equation (38).

(38) $$A_{aperture}=27.2\frac{t_{a}}{l_{a}}\;$$

where *l_a_* is the largest linear dimension of the cross-section of the aperture and *t_a_* is the depth of the aperture (length of “waveguide”).

As shown in Table 3, samples #1–#7 are considered to be thin in a specific analyzed frequency range 30 MHz–1.5 GHz for 7*δ*, 5*δ,* and also 3*δ* (with the exception of the most electrically conductive sample #2 in the frequency range 1.43–1.5 GHz), and therefore *A_aperture_* is not included in the *SE_aperture_* calculation. *K_aperture_* takes into account the geometrical dimensions of the aperture in a shielding barrier. It is described as [28], Equation (39).

(39) $$K_{aperture}=20\cdot \log\left(1+\ln\left(\frac{\ell }{s}\right)\right)\;$$

Equation (39) clearly shows the square apertures, i.e., *l* = *s*, do not influence *SE_aperture_*. Therefore, the resultant *SE_aperture_* is calculated for #1–#7, characterized as thin material, as shown in Equation (40).

(40) $$SE_{aperture}=R_{aperture}=158.55-20\cdot \log\left(\ell \cdot f\right)-20\cdot \log\left(\sqrt{n}\right)\;$$

### 6.2. Comparison of Equations for SE Fabric 

As previously mentioned, the *SE_fabric_* is calculated as Equation (35) or Equation (34) for #1. A similar equation was also previously mentioned as Equation (3) for metallized fabric shields [25,28]. Equation (3) is furthermore derived in order to compare Equations (3) and (34) for specific samples. The constant *C* = 0.129 is obtained in Equation (41) as:

(41) $$Cl\sqrt{f}=e^{-3}=>C=\frac{e^{-3}}{l\sqrt{f}}=\frac{2.71^{-3}}{0.389}=0.129\;$$

The authors in [25] use the value 2.71 for the mathematical constant *e*, which is approximately equal to e = 2.718 828. Moreover, the value of *l*$\sqrt{f}$ is equal to *l*$\sqrt{f}$ = 0.389, which is written as *l*$\sqrt{f}$ = 0.398 in [25] (and obviously calculated as *l*$\sqrt{f}$ = 0.389). The value of *l*$\sqrt{f}$ = 0.389 is calculated with respect to the description of Equation (26) from Equations (42)–(44).

(42) $$20\cdot \log\left(\frac{1}{4}\sqrt{\frac{\sigma }{\omega \mu_{r}\varepsilon_{0}}}\right)=100-20\cdot \log\left(\ell f\right)\;$$

(43) $$\log\left(\frac{\sigma }{32\pi f\mu_{r}\varepsilon_{0}}\right)=10-\log\left(\ell^{2}f^{2}\right)\;$$

(44) $$\log\left(\frac{\sigma }{32\pi \mu_{r}\varepsilon_{0}}\right)-10=-\log\left(\frac{1}{f}\right)-\log\left(\ell^{2}f^{2}\right)\;$$

Considering the electrical conductivity of copper, i.e., *σ* = 5.85 × 10^7^ S/m [38], the material used for electrically conductive textile material production in [25], Equation (44) is rewritten as shown in Equations (45)–(47).

(45) $$16.818-10=\log\left(\frac{1}{\ell^{2}f^{}}\right)\;$$

(46) $$10^{-6.818}=\ell^{2}f\;$$

(47) $$\ell \sqrt{f}=10^{\frac{-6.818}{2}}=10^{-3.409}=389.9\cdot 10^{-6}\;$$

The order of the value *l*$\sqrt{f}$ = 389.9 × 10^−6^ is multiplied by 1000 because of the units [mm] and [MHz] that are used in [25], i.e., 10^−3^ [m] and 10^6^ [Hz].

Equations (42)–(44) with no apertures are considered, and the condition *Z_M_* << *Z*_0_ is applied. The value 100 is derived from the *R_aperture_* equation, i.e., Equations (14)–(19), as shown in Equations (48)–(50).

(48) $$R_{aperture}=20\cdot \log\left(\frac{c}{x}\right)-20\cdot \log\left(\ell \cdot f\right)=100-20\cdot \log\left(\ell \cdot f\right)\;$$

(49) $$20\cdot \log\left(\frac{c}{x}\right)=100=>10^{5}=\frac{c}{x},$$

(50) $$x=\frac{c}{10^{5}},$$

The Equations (17)–(19) show the value of parameter *x* is equal to 2 (slot aperture), 2π (circular aperture), or 2$\sqrt{\pi }$ (square aperture). If the speed of light *c* = 3 × 10^8^ m/s is considered, the *x* is equal to *x* = 3000. Nevertheless, if the speed of light is equal to *c* = 186,000 miles/s, the *x* = 1.86. This result is close to the value, which is valid for the slot aperture. If the value *x* = 2 is used, Equation (48) is described as:

(51) $$R_{aperture}=20\cdot \log\left(\frac{186000}{2}\right)-20\cdot \log\left(\ell \cdot f\right)=99.4-20\cdot \log\left(\ell \cdot f\right),$$

The value 99.4 presented in Equation (51) is further rounded to the value 100.

The derivation of Equation (3) clearly shows the used equation is valid for copper metallized fabric, i.e., fabric without apertures, as the authors present in [25,28], and not valid for electrically conductive woven textile materials manufactured from the electrically conductive mixed and coated yarns.

## 7. Results and Discussion

The presumption of validity of *Z_M_* << *Z*_0_ and *σ* >> *ωε* for reflection loss of foil evaluation is presented in detail in chapter 3. It is also shown in Figure 1, Figure 2, Figure 3, Figure 4 and Figure 5, and Table 2. The validity of presented inequalities is based on a ratio of magnitudes of individual values, i.e., at least two orders of values of magnitude are required. As a result, a simplified version of the *Z*_M_, i.e., *σ* >> *ωε* is valid, and Equation (7) can be used for #1–#6 up to 10 GHz and for #7 up to approximately 6.9 GHz. The presumption of *Z_M_ << Z*_0_ is valid for #7, #6, and #4 up to 70 MHz, 439 MHz, and 1.8 GHz, respectively, if the validity of *σ* >> *ωε* is assumed and up to 34.6 MHz, 219.5 MHz, and 899.9 MHz, respectively, if the validity of *σ* >> *ωε* is not assumed. It can be seen that the greater the value of electrical conductivity of the samples is, the greater is the frequency limit that can be obtained. As a result, #1–#3 and #5 fulfill this validity up to the frequency limit, which is greater than 1.8 GHz (#4, i.e., *σ* = 1000 S/m). This frequency limit is chosen with respect to the limits of ASTM D4935-10, i.e., 0.03–1.5 GHz. The presumption of validity of *Z_M_* << *Z*_0_ and *σ* >> *ωε* is also verified for the *R_foil_* parameter, i.e., *Z_M_ << Z*_0_ and *σ* >> *ωε* are/are not considered (in all four combinations), and also for all samples #1–#7, Figure 5. It shows the greatest difference is reached for the sample with the lowest electrical conductivity from #1–# 5, i.e., #4, and it is equal to about 0.5 dB in 10 GHz. Similar results are obtained for #6 and #7, i.e., 1 dB and 2 dB, respectively, in 10 GHz. As a consequence, the presented limits for *Z_M_ << Z*_0_, e.g., 70 MHz, 439 MHz, and 1.8 GHz for #7, #6, and #4, respectively, can be ignored and the relevant error has to be taken into account.

Derivation of reflection loss of one aperture shows an importance of determination of the effective aperture *A*_e_ for different shapes of apertures. It is clear that knitted fabrics require a different calculation of reflection loss for one aperture in comparison with woven fabrics.

Calculation of reflection loss of multiple apertures is not unified in the scientific literature [32,36,37] because different conditions for calculation of the number of apertures *n* are presented. It is, for instance, the calculation of the total length of linear array of apertures *l_c_*. Obviously, different sizes of samples result in different values of total length of linear arrays of apertures *l_c_*. We consider standard ASTM D4935-10, which is one of the most used standards for SE evaluation, and *l_c_* = 0.069 m, Figure 7. This parameter has to be less than ½ of the wavelength, and it fulfills this condition in the frequency range defined in ASTM D4935-10, i.e., 0.03–1.5 GHz, Figure 6. One of the conditions is also that the material has to be thin, which is verified by comparison of the equivalent depth of penetration *δ*, usually 3*δ*, and the thickness of the material *t*, i.e., material is considered to be thin if inequality 3*δ > t* is valid. The results show the penetration depth 3*δ* is greater than the thickness of #2 (the sample with the highest value of electrical conductivity, i.e., the value of penetration depth is the lowest from all samples) in the frequency range 0.03–1.43 GHz, Figure 8 and Table 3. The penetration depth for 5*δ* and 7*δ* is also analyzed in order to verify whether there are any reflections from the “far” interface of the material for the frequency beyond 1.43 GHz, i.e., the material can be considered to be thin. The results show it is valid for 5*δ* and 7*δ* in the frequency ranges 0.03–3.98 GHz, and 0.03–7.79 GHz, respectively, Figure 8 and Table 3. The results of reflection loss of multiple apertures evaluation show (20) has to be considered in reflection loss calculations and the values of electrical conductivity, and thickness of samples has to be considered because of thin/thick material evaluation.

Evaluation of the SE fabric considers a simplified version of the reflection loss of foil, i.e., *Z_M_* << *Z*_0_ and *σ* >> *ωε* are valid, a boundary condition, which defines a decrease of the amplitude by about 95% in specific materials, i.e., equivalent of 3 depth of penetration e^−3^, and number of apertures, which is calculated with respect to the longest linear array of apertures of ASTM 4935-10, i.e., *l_c_* = 0.069 m. As a result, constant *C*, the value in the exponent of Euler’s number in the equation of the SE fabric calculation, is derived in Equation (32), Table 4. It clearly shows the SE fabric evaluation depends on sett, number of the longest linear array of apertures, and electric conductivity of each sample. As a consequence, the equation for SE calculation of woven fabrics manufactured from the electrically conductive mixed and coated yarns with square apertures is generally derived by Equation (35) with respect to Equation (32).

Individual components of SE fabric evaluation are *SE_foil_*, i.e., the SE values for metallic foil of the same thickness as the fabric Equations (4) and (36), which is derived and described in many research papers [24,26,27,28,29,30], and *SE_aperture_*, i.e., the SE values for metallic foil of the same thickness as the fabric with aperture(s), which is derived in this paper for particulate-blended shielding electrically conductive textile composites, i.e., woven fabrics manufactured from the electrically conductive mixed and coated yarns with square apertures, samples #1–#7. Calculation of the reflection loss of aperture *R_aperture_* is a sum of reflection loss of one aperture *R_aperture_square_* (Equation (17)) and reflection loss of multiple apertures *R_aperture_multiple_* (Equation (20)), i.e., Equation (37). The absorption loss *A_aperture_* is neglected because the material is considered to be thin. A correction of geometrical dimensions of the aperture *K_aperture_* does not influence *SE_aperture_* because of square apertures. As a result, the resultant *SE_aperture_* is equal to *R_aperture_*.

Derivation of *SE_aperture_* (Equation (40)) and *SE_fabric_* (Equation (35)) clearly shows many factors have to be considered, i.e., shape of apertures, thickness of fabric in comparison with penetration depth (in order to determine conditions for thin/thick material), values of electrical conductivity, validation of *Z_M_* << *Z*_0_ and *σ* >> *ωε*, total length of linear array of apertures, and sett of the fabric. It also shows (3) is valid for copper metallized fabric, i.e., fabric without apertures, and not valid for electrically conductive woven textile materials manufactured from the electrically conductive mixed and coated yarns. 

**Figure 9:**
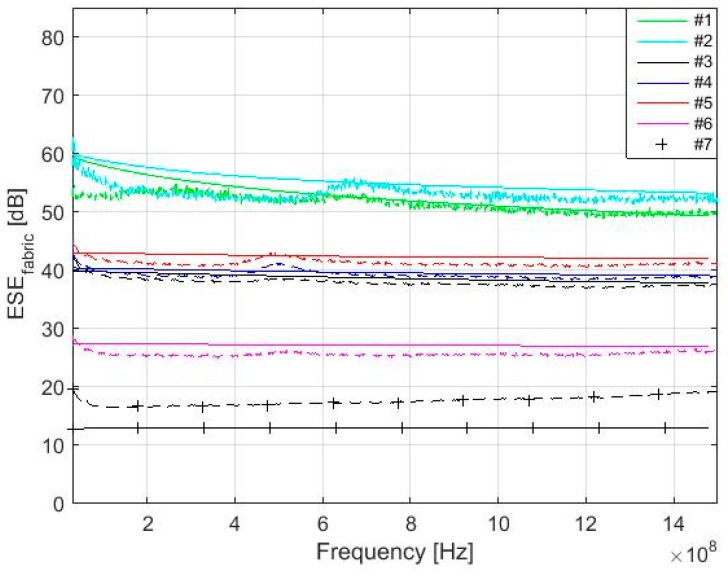
Comparison of the modeling (solid line) and measurement results for #1–#7 in 30 MHz–1.5 GHz.

Modeling of the *SE_fabric_* (Equation (35)) with respect to used textile material, i.e., electrical conductivity of samples described in Table 1, evaluation of the constant *C* (Equation (32)) and *n* (Equation (33)) shown in Table 4, calculation of the *SE_foil_* presented in Equation (4) and specified in Equation (7), and *SE_aperture_* derived in Equation (40) can be performed and compared with measurement results, Figure 9.

**Figure 10:**
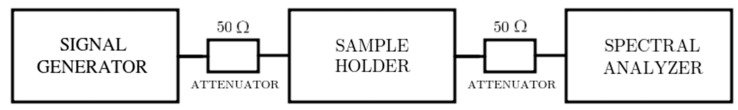
Schematic block diagram of experimental setup according to ASTM D4935-10.

**Figure 11:**
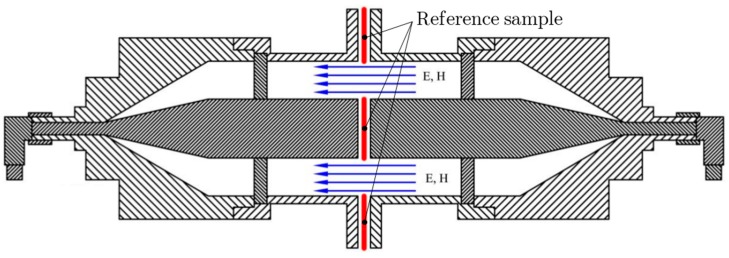
Cross section of sample holder with reference sample according to ASTM D4935-10.

**Figure 12:**
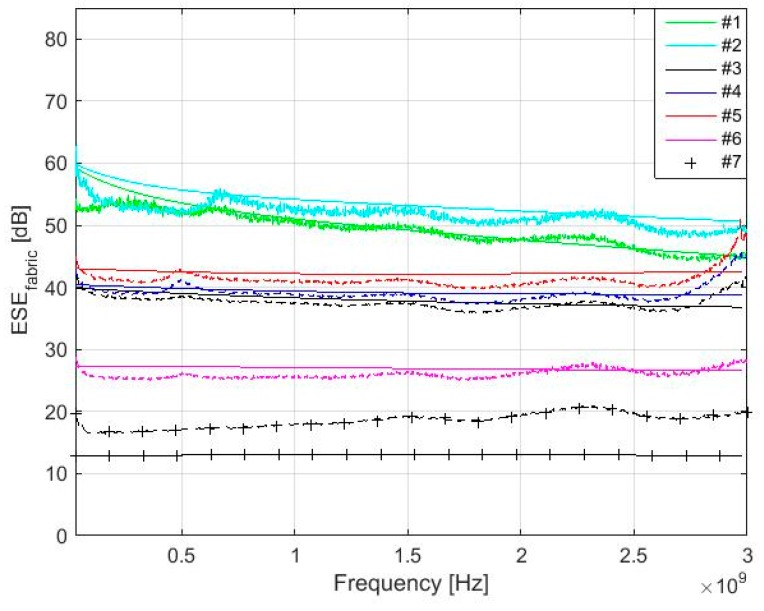
Comparison of the modeling (solid line) and measurement results for #1–#7 in 30 MHz–3 GHz.

**Figure 13:**
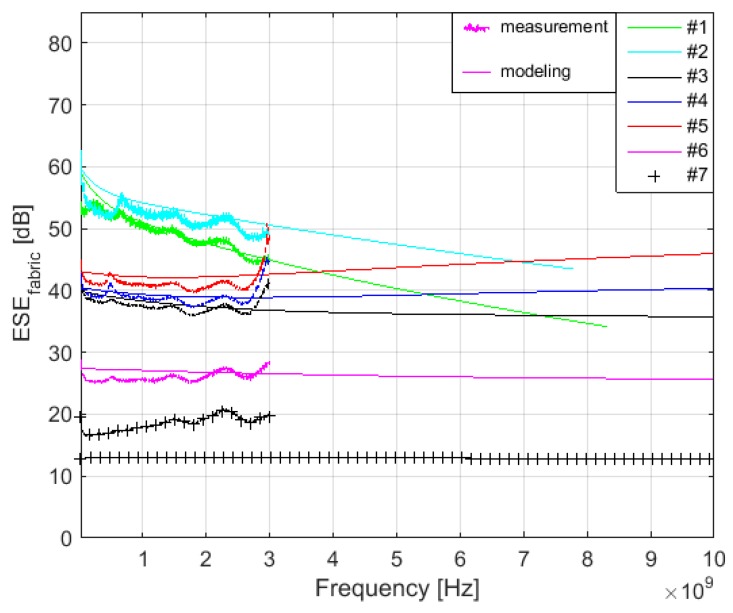
Comparison of the modeling (solid line) and measurement results for #1–#7 in 30 MHz–3 GHz (7*δ*) and modeling (solid line) results in 3 GHz–10 GHz.

Measurement is performed according to ASTM D4935-10 [39] (22 °C, RH 48%). A schematic block diagram of the experimental setup is shown in Figure 10 and a cross section of the sample holder with reference sample is shown in Figure 11. The sample holder is an enlarged coaxial transmission line with special taper sections to maintain a characteristic impedance of 50 Ω throughout the entire length of the sample holder. The reference sample is intended for calibration of the measurement setup. The load sample causes the loss of the passing high-frequency signal, which can be recorded by spectral analyzer. The results show the presented equations are valid for electrically conductive textile materials with a value of electrical conductivity equal to and higher than *σ* = 244 S/m, i.e., samples #1–#6. The maximal difference between modeling and measurement results was obtained for #1 and #2 in the frequency range 30–280 MHz. This is in the range of 2–6 dB. Nevertheless, it is within the random error of the used measurement method, which is defined in ASTM D4935-10 as ±5 dB. It is also within an observed standard deviation based on measurements by five laboratories on five samples presented in ASTM D4935-10 as 6 dB [39]. It is important to note that the measurement results are evaluated with respect to ASTM D4935-10, which defines a test procedure in the frequency range 0.03–1.5 GHz. The measurement results are therefore only informative in the frequency range 1.5 GHz–3 GHz, i.e., increasing value of the measured SE is caused by the excitation of modes other than the transverse electromagnetic mode (TEM), Figure 12. The results for sample #7 show these equations have to be modified for other materials (*σ* = 39 S/m). The frequency range of the model can also be extended, Figure 13. It shows both an increasing and decreasing trend of the *SE_fabric_* of samples.

## 8. Conclusions

This paper is focused on a derivation of a numerical model of electromagnetic shielding effectiveness for woven fabrics manufactured from electrically conductive mixed and coated yarns. Commonly used measurement techniques are mentioned. Basic equations of electromagnetic shielding effectiveness calculations are presented.

An evaluation of reflection loss of foil is described in detail and verifies the assumption of *Z_M_ << Z*_0_ and *σ* >> *ωε* in a frequency range up to 10 GHz is valid for samples with electrical conductivity higher than 1000 S/m with an error up to 0.5 dB, for a sample with electrical conductivity 244 S/m with an error not exceeding 1 dB, and for a sample with electrical conductivity 39 S/m and an error up to 2 dB.

A derivation of reflection loss of one aperture is performed for slot, square, and circular apertures. The evaluation of reflection loss of multiple apertures describes the different calculation of the number of apertures in a shielding barrier and verifies presented conditions for its calculation. The longest linear array of apertures is used for the numerical model. 

A complete derivation of electromagnetic shielding effectiveness of woven fabrics manufactured from the electrically conductive mixed and coated yarns is presented in detail. It shows the equations for electromagnetic shielding effectiveness evaluation differ for materials that are considered to be thin or thick (based on penetration depth and thickness comparison), for different values of electrical conductivity, and for different setts used in the manufacturing process. 

A comparison of modeling and measurement results of electromagnetic shielding effectiveness fabric is performed in the frequency range 0.03–1.5 GHz according to ASTM D4935-10. The results clearly show a numerical model is valid for electrically conductive woven textile materials with a value of electrical conductivity equal to and higher than *σ* = 244 S/m. The results of the numerical mode are also extended up to 10 GHz in order to show the trend of electromagnetic shielding effectiveness.

## Figures and Tables

**Table 1:** Fabric specifications.

No.	Composition	Fabric Structure	Mass per Unit Area [g/m^2^]	Warp/Weft Density *d*_w_ [t/cm]	Linear Density [tex]	Electrical Conductivity [S/m]	Standard Deviation [S/m]
1	SilveR.STAT^®^ 240dtex/10F	Plain weave	75	13/13	24	1.71 × 10^4^	9.72 × 10^2^
2	SilveR.STAT^®^ 240dtex/10F	Plain weave	95	16/16	24	1.77 × 10^4^	5.69 × 10^2^
3	60% PES/40% SilveR.STAT^®^ 3.3dtex	Plain weave	92	13/13	29.5	1.07 × 10^3^	2.32 × 10^1^
4	60% PES/40% SilveR.STAT^®^ 3.3dtex	Plain weave	115	16/16	29.5	1.00 × 10^3^	3.29 × 10^1^
5	60% PES/40% SilveR.STAT^®^ 3.3dtex	Plain weave	135	19/19	29.5	1.37 × 10^3^	5.14 × 10^1^
6	40% PES/60% SilveR.STAT^®^ 1.7dtex	Plain weave	115	16/16	29.5	2.44 × 10^2^	8.99 × 10^0^
7	60% PES/40% SilveR.STAT^®^ 1.7dtex	Plain weave	117	16/16	29.5	3.9 × 10^1^	2.13 × 10^0^

**Table 2:** Results of ratio of the *σ* and *ωε* evaluation.

	Sample #6	Sample #7
Frequency [GHz]	*σ*/*ωε*	*σ*/*ωε*
1.5	2924	461.4
3	1462	230.7
4	1097	173
10	438.6	69.21

**Table 3:** Material characterization and thin material evaluation*.*

#	Warp/Weft Density *d*_w_ [t/cm]	Fabric Thickness [µm]	Yarn Diameter [µm]	Aperture Length [µm]	Thin Material 7*δ* [GHz]	Thin Material 5*δ* [GHz]
**1**	13/13	295	251	517	0.03–8.34	0.03–4.25
**2**	16/16	300	251	373	0.03–7.79	0.03–3.98
**3**	13/13	469	239	530	0.03–52	0.03–27
**4**	16/16	537	239	386	0.03–43	0.03–22
**5**	19/19	533	239	287	0.03–31	0.03–16
**6**	16/16	476	220	405	0.03–228	0.03–110
**7**	16/16	491	240	385	0.03–1316	0.03–680

**Table 4:** Calculation of the *C* constant.

#	*σ* [S/m]	*d*_w_ [t/cm]	*n*	*C*
1	1.71 × 10^4^	13	89	2.44 × 10^−2^
2	1.77 × 10^4^	16	110	2.76 × 10^−2^
3	1.07 × 10^3^	13	89	6.1 × 10^−3^
4	1.00 × 10^3^	16	110	6.6 × 10^−3^
5	1.37 × 10^2^	19	131	8.4 × 10^−3^
6	2.44 × 10^2^	16	110	3.2 × 10^−3^
7	3.9 × 10^1^	16	110	1.3 × 10^−3^
